# Long‐Term Prognostic and Hemodynamic Outcomes of Intensive Immunosuppressive Therapy in Patients With Pulmonary Arterial Hypertension Associated With Connective Tissue Disease

**DOI:** 10.1111/1756-185X.70431

**Published:** 2025-10-09

**Authors:** Kaito Yamada, Nobuhiro Yaoita, Taijyu Satoh, Saori Yamamoto, Yusuke Yamada, Naoki Chiba, Kohei Komaru, Haruka Sato, Nobuhiro Kikuchi, Hideaki Suzuki, Kotaro Nochioka, Shunsuke Tatebe, Satoshi Miyata, Tomonori Ishii, Satoshi Yasuda

**Affiliations:** ^1^ Department of Cardiovascular Medicine Tohoku University Graduate School of Medicine Sendai Japan; ^2^ Teikyo University Graduate School of Public Health Tokyo Japan; ^3^ Department of Hematology and Rheumatology Tohoku Medical and Pharmaceutical University Hospital Sendai Japan

**Keywords:** intensive immunosuppressive therapy, PAH associated with connective tissue disease, systemic sclerosis

## Abstract

**Background:**

Intensive immunosuppressive therapy (IIT) is recommended for PAH associated with connective tissue disease (CTD‐PAH). However, the long‐term effects of IIT on pulmonary hemodynamics in this population remain unexplored. Additionally, its effectiveness in patients with systemic sclerosis (SSc)‐associated PAH (SSc‐PAH) is poorly understood.

**Methods and Results:**

This retrospective analysis included 69 consecutive patients with CTD‐PAH treated at our institution (men/women: 9/60, mean age 55.3 ± 14.0 years). Patients were divided into two groups, wherein 41 patients received IIT (IIT group) and 28 did not (non‐IIT group). Both groups received conventional vasodilator therapy. The prognosis and pulmonary hemodynamics were evaluated in all patients. The IIT group exhibited significantly lower rates of PAH‐related mortality (*p* < 0.001) compared with the non‐IIT group. The mean PAP (mPAP) improved significantly in the IIT group during the follow‐up (baseline: 38.7 ± 12.2 mmHg; 1 year: 27.0 ± 8.2 mmHg; 5 years: 26.8 ± 7.3 mmHg, *p* < 0.05), while it remained unchanged in the non‐IIT group. None of the patients with CTD‐PAH required IIT retreatment. Among the 27 patients with SSc‐PAH, the IIT group (*n* = 9) showed a significantly greater improvement in mPAP compared with the non‐IIT group (*n* = 18) (ΔmPAP at 1 year: −13.4 ± 6.5 mmHg in IIT group vs. −3.0 ± 6.2 mmHg in non‐IIT group, *p* < 0.001).

**Conclusions:**

This study's findings suggest that IIT may lead to sustained improvements in pulmonary hemodynamics and better long‐term outcomes in patients with CTD‐PAH, including potential benefits in those with SSc‐PAH.

**Abbreviations**
BNP: brain natriuretic peptide
CI: cardiac index
CTD: connective tissue disease
ERA: endothelin receptor antagonist
IIT: intensive immunosuppressive therapy
ILD: interstitial lung disease
MCTD: mixed connective tissue disease
PAH: pulmonary arterial hypertension
PAP: pulmonary artery pressure
PAWP: pulmonary arterial wedge pressure
PDE5i: phosphodiesterase inhibitor
PGI2: prostacyclin
PVR: pulmonary vascular resistance
RAP: right arterial pressure
sGC: soluble guanylate cyclase
SjS: Sjögren syndrome
SLE: systemic lupus erythematosus
SSc: systemic sclerosis
SvO_2_
: mixed venous oxygen saturation

**Summary**

- PAH‐related death was significantly lower in patients with CTD‐PAH who received IIT (IIT group) than in those who did not receive IIT (non‐IIT group).
- Pulmonary hemodynamics of CTD‐PAH including SSc‐PAH were significantly improved in the IIT group but remained unchanged in the non‐IIT group over a median follow‐up period of 5.3 years.
- IIT might have potential effectiveness in patients with SSc‐PAH whose pulmonary hemodynamics was improved with acute vasoreactivity test.

## 1 Introduction

Pulmonary arterial hypertension (PAH) is a life‐threatening condition caused by obstruction of the small pulmonary arteries due to vascular remodeling [1, 2, 3]. It is defined as a mean pulmonary arterial pressure (mPAP) > 20 mmHg at rest and pulmonary vascular resistance (PVR) exceeding 160 dyne s/cm^5^ [4]. Among the various subtypes of pulmonary hypertension (PH), PAH associated with connective tissue disease (CTD‐PAH) has a poor prognosis, partly owing to its recalcitrance to conventional vasodilator therapy [5]. Immunological and inflammatory mechanisms are implicated in the progression of PAH in patients with CTD [6]. Therefore, the 2022 European Society of Cardiology/European Respiratory Society (ESC/ERS) guidelines recommend the addition of immunosuppressive therapy to pulmonary vasodilator therapy for the treatment of CTD‐PAH [4].

Intensive immunosuppressive therapy (IIT), which typically includes glucocorticoids and cyclophosphamide, has been shown to improve symptoms, pulmonary hemodynamics, and prognosis in patients with PAH associated with systemic lupus erythematosus (SLE), mixed connective tissue disease (MCTD), and Sjögren syndrome (SjS) [7, 8, 9]. However, these studies were limited by short follow‐up periods and the investigation of only the prognosis, rather than the long‐term pulmonary hemodynamics, leaving the long‐term effects of IIT on pulmonary hemodynamics unclear. Furthermore, since previous studies indicated that IIT may not be effective in patients with PAH associated with systemic sclerosis (SSc) [4, 9], the ESC guidelines advised against IIT for patients with SSc‐PAH [4]. However, these studies were small, engendering uncertainty about the effectiveness of IIT for all patients with SSc‐PAH.

Therefore, the present study aimed to assess the long‐term effectiveness of IIT on pulmonary hemodynamics and prognosis in patients with CTD‐PAH and to examine its effect in patients with SSc‐PAH.

## 2 Material and Methods

This study was approved by the Institutional Review Board of Tohoku University, Sendai, Japan, and the Medical Ethics Review Committee (approval no. 2021‐1‐208), which waived the requirement for informed consent owing to its retrospective design. The opt‐out method was used to obtain patient consent for participation in this study.

### 2.1 Cohort of PH Patients

**FIGURE 1:**
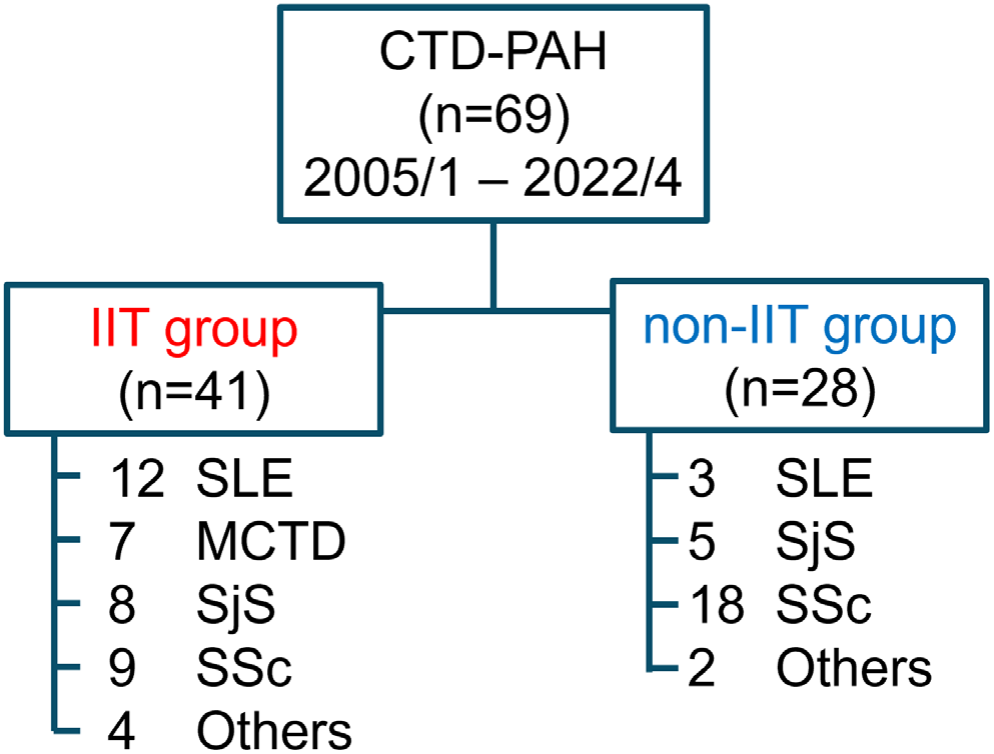
Enrollment algorithm. In our pulmonary hypertension cohort, 69 patients were diagnosed with connective tissue disease‐associated pulmonary arterial hypertension (CTD‐PAH) between 2005 and 2022. Among them, 41 consecutive patients received intensive immunosuppressive therapy (IIT) in conjunction with conventional vasodilator therapy (IIT group), whereas 28 patients received only conventional vasodilator therapy (non‐IIT group). CTD, connective tissue disease; MCTD, mixed connective tissue disease; PAH, pulmonary arterial hypertension; SjS, Sjögren's syndrome; SLE, systemic lupus erythematosus; SSc, systemic sclerosis.

The cohort comprised 69 consecutive patients with CTD‐PAH who underwent right heart catheterization (RHC) at Tohoku University Hospital between 2005 and 2022 (Figure 1) [8, 10]. These patients were regularly followed up every 6–12 months with RHC [8, 10].

### 2.2 Diagnosis of PH Subtypes

All 69 patients underwent RHC, with PH defined as an mPAP ≥ 25 mmHg at rest [11]. The diagnosis of PAH was contingent upon an additional criterion of pulmonary arterial wedge pressure ≤ 15 mmHg [11]. Acute pulmonary vasoreactivity testing was performed during right heart catheterization (RHC), where nitric oxide (NO) was inhaled for 5 min to assess changes in pulmonary hemodynamics [12]. A positive response to the acute pulmonary vasoreactivity test was defined as a reduction in mean pulmonary artery pressure (mPAP) ≥ 10 mmHg and mPAP ≤ 40 mmHg, with either an increased or unchanged cardiac output [4]. CTD‐PAH and liver disease‐associated PAH were diagnosed clinically and through blood tests, according to established criteria [4]. We defined SLE, MCTD, SjS, and SSc based on each of the classification criteria [13, 14, 15, 16]. Overlap syndrome was defined as meeting classification criteria for two connective tissue diseases. Patients with overlap syndrome involving systemic sclerosis (SSc) were classified as having SSc‐PAH. All connective tissue diseases were diagnosed by rheumatologists using established criteria [13, 14, 15, 16]. Congenital heart disease‐associated PH was identified via echocardiography, whereas chronic thromboembolic PH was diagnosed using ventilation‐perfusion scans and computed tomography (CT). Lung disease and hypoxia were diagnosed using pulmonary function testing, arterial blood gas analysis, chest radiography, and CT. Patients without these abnormalities were diagnosed with idiopathic PAH [4].

### 2.3 Data Collection

Baseline characteristics, such as age, sex, height, weight, use of pulmonary vasodilators, clinical diagnosis, comorbidities (CTDs, interstitial lung disease), plasma B‐type natriuretic peptide levels, and pulmonary hemodynamic data from catheterization before and after acute pulmonary vasoreactivity testing [12] were extracted from the medical records. The pulmonary hemodynamic parameters included pulmonary arterial wedge pressure, pulmonary artery pressure (PAP), right atrial pressure, cardiac output, cardiac index (CI), PVR, and mixed venous oxygen saturation. All‐cause mortality and PH‐related mortality (defined as lung transplantation or death due to worsening of right heart failure) were investigated in each patient. Safety outcomes during the follow‐up period were also investigated. Infection was defined as the number of patients requiring hospitalization for treatment. Severe infection was defined as the number of patients whose infection resulted in death. Malignancy was defined as newly diagnosed malignant tumors during the follow‐up period.

### 2.4 IIT for CTD‐PAH

The IIT regimen consisted of cyclophosphamide (500 mg intravenous, 10 times over 1 year, administered monthly for the first 3 months, and once every 1–3 months thereafter) combined with glucocorticoids (1 mg/kg/day per oral for the first month, with gradual tapering to 5–10 mg/day every 2–4 weeks until a maintenance dose of 5–10 mg/day was reached) [8, 10]. RHC was performed at baseline and every 6–12 months during the follow‐up [8, 10].

### 2.5 Statistical Analysis

The data were presented as the mean ± SD for normally distributed continuous variables, and as medians with interquartile ranges (25th–75th percentiles) for continuous variables with non‐normal distribution and categorical variables. Paired *t*‐tests were used to compare continuous variables, and Fisher's exact test was used for categorical variables. Survival outcomes, including all‐cause mortality, cardiovascular mortality, and lung transplantation, were estimated using the Kaplan–Meier method; the differences between the survival curves were assessed using the log‐rank test. The association between groups and time‐to‐event outcomes was assessed using Cox regression analysis adjusted for age and sex. Similar analyses were conducted for PH‐related death in each group. All statistical analyses were conducted using GraphPad Prism 9.0E (GraphPad Software Inc., La Jolla, CA, USA). *p* values < 0.05 were considered statistically significant.

## 3 Results

### 3.1 Study Population and Baseline Characteristics

This study enrolled 69 consecutive patients with CTD‐PAH who underwent RHC between 2005 and 2022. Forty‐one patients received IIT in conjunction with conventional vasodilator therapy (IIT group), while 28 patients received conventional vasodilator therapy alone (non‐IIT group) (Figure 1). Among the non‐IIT group, 11 of 28 patients received maintenance doses of glucocorticoids or immunosuppressive agents as follows: prednisolone 1–5 mg/day (*n* = 5), prednisolone 6–10 mg/day (*n* = 4), mycophenolate mofetil 500 mg/day (*n* = 1), and tacrolimus hydrate 1 mg/day (*n* = 1). The median follow‐up period was 4.3 years. There were no differences in the study enrollment period between the groups. Although four patients in each group were lost in the follow‐up periods (Table S1), there were no differences in disease severity between these patients. The baseline clinical characteristics of the IIT and non‐IIT groups are presented in Table 1. Patients in the IIT group were younger than those in the non‐IIT group (49.1 ± 12.8 years vs. 64.3 ± 49.1 years, *p* < 0.001). In the IIT group, the proportion of patients with SLE and those with MCTD was higher and that of patients with SSc was lower compared to the non‐IIT group. Renal function was lower in the non‐IIT group, although no significant differences were evident in the baseline hemodynamic parameters between the groups (mPAP: 38.6 ± 12.0 mmHg [IIT] vs. 35.4 ± 8.1 mmHg [non‐IIT], *p* = 0.313; PVR: 678.7 ± 484.2 vs. 516.4 ± 256.3 dyne s/cm^2^, *p* = 0.389; Table 1).

**TABLE 1:** Baseline characteristics of patients with pulmonary arterial hypertension associated with connective tissue disease treated with and without intensive immunosuppressive therapy.

	IIT (*n* = 41)	Non‐IIT (*n* = 28)	*p*
Age (years)	49.1 ± 12.8	64.3 ± 10.4	< 0.001
Female (%)	38 (92.7)	22 (78.6)	0.087
Follow‐up period (month)	64.0 (30.0–119.5)	46.0 (31.3–66.0)	0.092
Underlying CTDs (%)			
SLE	12 (29.3)	3 (10.7)	0.067
MCTD	7 (17.1)	0 (0.0)	0.021
SjS	8 (19.5)	5 (17.9)	0.863
SSc	9 (30.0)	18 (64.3)	< 0.001
Others	4 (9.8)	2 (7.1)	0.705
Time from diagnosis of CTD to diagnosis of PAH	84.0 (0.0–132.0)	23.0 (0.0–114.0)	0.403
Simultaneous diagnosis of PAH and CTD	15 (36.6)	8 (30.8)	0.625
Baseline measurement			
Hb (g/dL)	12.7 ± 2.0	12.3 ± 1.9	0.456
Creatinine (mg/dL)	0.6 ± 0.3	1.1 ± 1.4	0.030
CRP (mg/dL)	0.0 (0.0–0.2)	0.0 (0.0–0.2)	0.597
BNP (pg/mL)	65.2 (12.5–280.0)	79.9 (29.2–207.0)	0.325
NYHA			
I	6 (14.6)	1 (3.6)	0.135
II	21 (51.2)	18 (65.3)	0.282
III	11 (26.8)	7 (25.0)	0.865
IV	3 (7.3)	2 (7.1)	0.978
Pulmonary hemodynamic			
mPAP (mmHg)	38.6 ± 12.0	35.4 ± 8.1	0.313
PVR (dyne s/cm^2^)	678.7 ± 484.2	516.4 ± 256.3	0.389
CI (L/min/m^2^)	2.5 ± 0.6	2.5 ± 0.4	0.886
PAWP (mmHg)	8.1 ± 3.3	8.3 ± 6.9	0.467
RAP (mmHg)	6.0 ± 3.8	4.7 ± 3.7	0.167
SvO_2_ (%)	66.5 ± 9.4	65.8 ± 8.8	0.362

*Note:* Continuous variables are expressed as the mean ± SD, except for the follow‐up period. The eGFR and BNP levels are expressed as medians with interquartile ranges. The parameters were compared using the Mann–Whitney *U* test.

Abbreviations: BNP, brain natriuretic peptide; CI, cardiac index; CTD, connective tissue disease; ILD, interstitial lung disease; MCTD, mixed connective tissue disease; PAH, pulmonary arterial hypertension; PAP, pulmonary artery pressure; PAWP, pulmonary arterial wedge pressure; PVR, pulmonary vascular resistance; RAP, right arterial pressure; SjS, Sjögren syndrome; SLE, systemic lupus erythematosus; SSc, systemic sclerosis; SvO_2_, mixed venous oxygen saturation.

### 3.2 Long‐Term Prognosis of CTD‐PAH

All CTD‐PAH patients in the IIT group received IVCY 10 times, with a cumulative cyclophosphamide dose of 5000 mg. During the follow‐up period, 14 CTD‐PAH patients died (7 in each group). In the IIT group, the causes of death were interstitial pneumonia in five patients, bacterial pneumonia in one patient, and PH‐related death in one patient. In the non‐IIT group, the causes of death were PH‐related in six patients and trauma in one patient.

**FIGURE 2:**
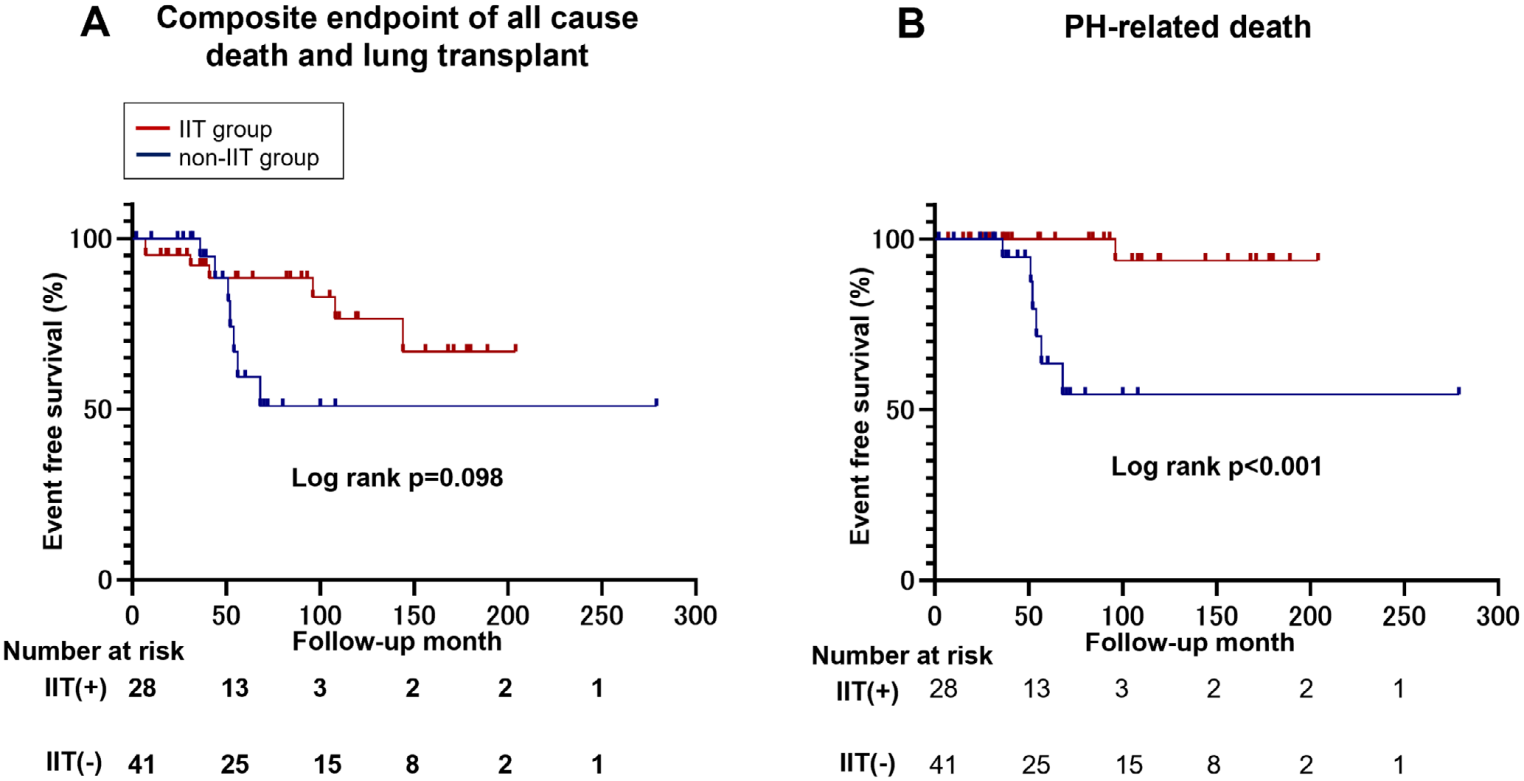
Effects of IIT on long‐term prognosis. (A) Kaplan–Meier curves for the composite endpoint of all‐cause mortality and lung transplantation. (B) Kaplan–Meier curves for pulmonary hypertension (PH)–related mortality. The curves compare patients treated with intensive immunosuppressive therapy (IIT group: *n* = 41) and conventional vasodilator therapy with those who received only conventional vasodilator therapy (non‐IIT group: *n* = 28) during the follow‐up period.

Patients in the IIT group demonstrated significantly better long‐term survival than those in the non‐IIT group (Figure 2A,B). Although the difference in all‐cause mortality did not attain statistical significance (*p* = 0.098), PH‐related mortality was significantly lower in the IIT group (*p* < 0.001). The 1‐, 3‐, 5‐, and 10‐year survival rates in the IIT group were 95.0%, 91.9%, 88.3%, and 76.7%, respectively, compared with 96.0%, 91.0%, 63.2%, and 54.3%, respectively, in the non‐IIT group. An adjusted Cox regression analysis for age and sex showed that the IIT group tended to have a lower risk of PH‐related death compared with the non‐IIT group (hazard ratio [HR] 0.09; 95% CI 0.01–1.36; *p* = 0.082). After further adjustment for age, sex, the presence of systemic sclerosis, and NYHA functional class, IIT was associated with a significantly lower risk of PH‐related death (HR 0.02; 95% CI 0.00–0.63; *p* = 0.027). These findings indicate that IIT is associated with improved long‐term prognosis in patients with CTD‐PAH, as reported previously [7].

### 3.3 Pulmonary Hemodynamics of CTD‐PAH

**FIGURE 3:**
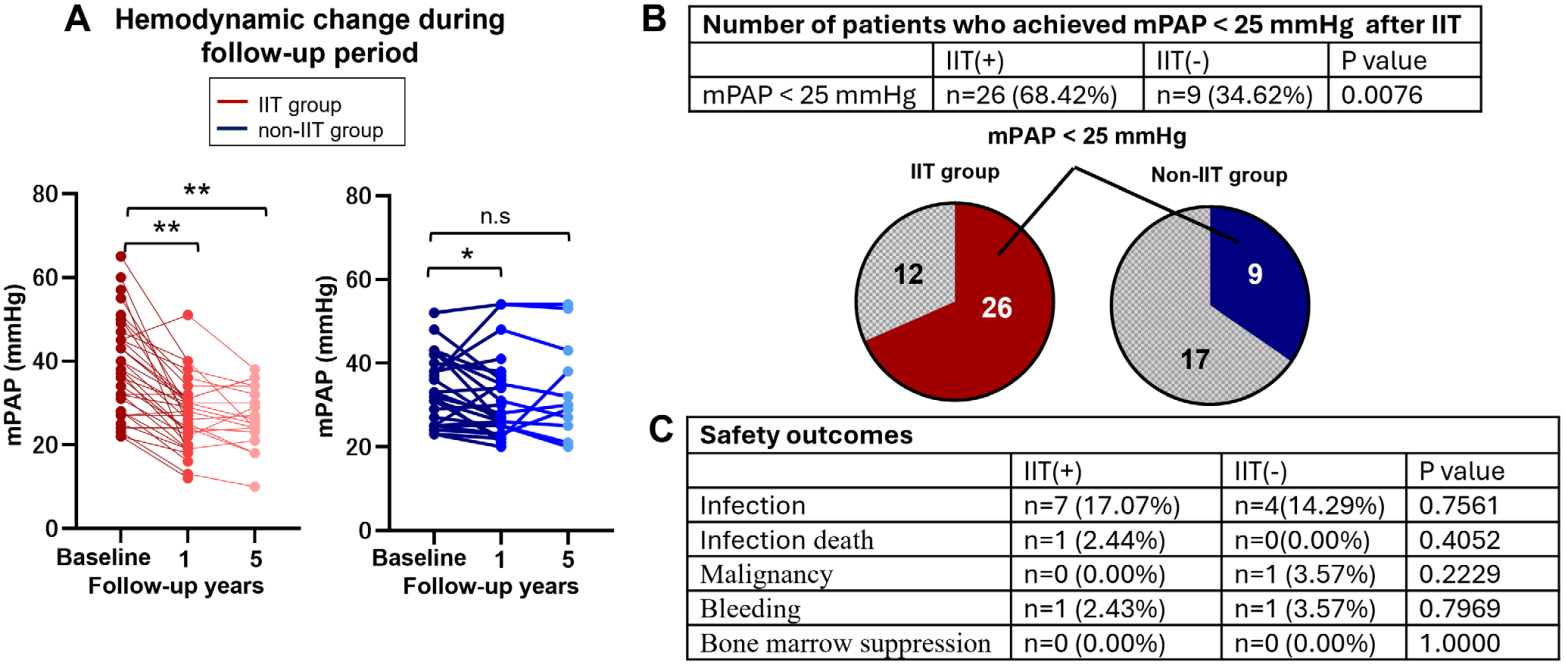
Effects of IIT on pulmonary hemodynamics. (A) Effect of intensive immunosuppressive therapy (IIT) on pulmonary hemodynamics in patients with CTD‐PAH after 1 and 5 years. The red bars represent the IIT group, while blue bars represent the non‐IIT group. (B) Number of patients who achieved a mean pulmonary artery pressure (mPAP) < 25 mmHg following intensive immunosuppressive therapy or conventional treatment during the follow‐up period. (C) Safety outcome during the follow‐up period. Infection was defined as the number of patients requiring hospitalization for treatment. Severe infection was defined as the number of patients whose infection resulted in death. Malignancy was defined as newly diagnosed malignant tumors. Bleeding was defined as BARC type 3 or higher. Bone marrow suppression was defined as the occurrence of grade 3 or higher hematologic adverse events in accordance with the Common Terminology Criteria for Adverse Events (CTCAE) version 4.0 during the follow‐up period. The results are presented as mean ± SEM. Statistical comparisons were performed using the paired *t*‐test, two‐tailed Student's *t*‐test, or Welch's *t*‐test, as appropriate. The significance levels are as follows: *p < 0.05, **p < 0.01. CTD‐PAH, pulmonary arterial hypertension associated with connective tissue disease.

Next, we examined the changes in pulmonary hemodynamics during the follow‐up period (baseline, 1 year, and 5 years: *n* = 69, 61, 30, respectively). In the non‐IIT group, the mPAP initially improved at 1 year but increased again by year 5 (baseline, 1 year, and 5 years: 35.4 ± 8.1, 31.5 ± 9.5, 33.6 ± 12.0 mmHg, respectively). In contrast, the mPAP in the IIT group showed significant improvement throughout the follow‐up period (baseline, 1 year, and 5 years: 38.6 ± 12.0, 30.0 ± 8.2, 26.8 ± 7.3 mmHg, respectively, *p* < 0.001) (Figure 3A). Additionally, a significantly higher proportion of patients in the IIT group achieved an mPAP < 25 mmHg during the follow‐up period than those in the non‐IIT group (68.4% vs. 34.6%, *p* = 0.008) (Figure 3B) [17], Furthermore, more patients in the IIT group avoided being administered the pulmonary vasodilators during the follow‐up period compared with the non‐IIT group (IIT group vs. the non‐IIT group: 12/41 [29.3%] vs. 2/28 [7.1%], *p* = 0.025). Notably, none of the patients in the IIT group required a second round of IIT due to worsening pulmonary hemodynamics during follow‐up, and there were no interruptions of IIT due to any adverse events. Furthermore, during the follow‐up period, the incidence of safety outcomes (infections, infection‐related mortality, newly diagnosed malignancy, bleeding, bone marrow suppression) did not increase in the IIT group (Figure 3C). These results suggest that IIT not only improves pulmonary hemodynamics but also maintains them in the long term in patients with CTD‐PAH.

### 3.4 IIT for SSc‐Associated PAH

**FIGURE 4:**
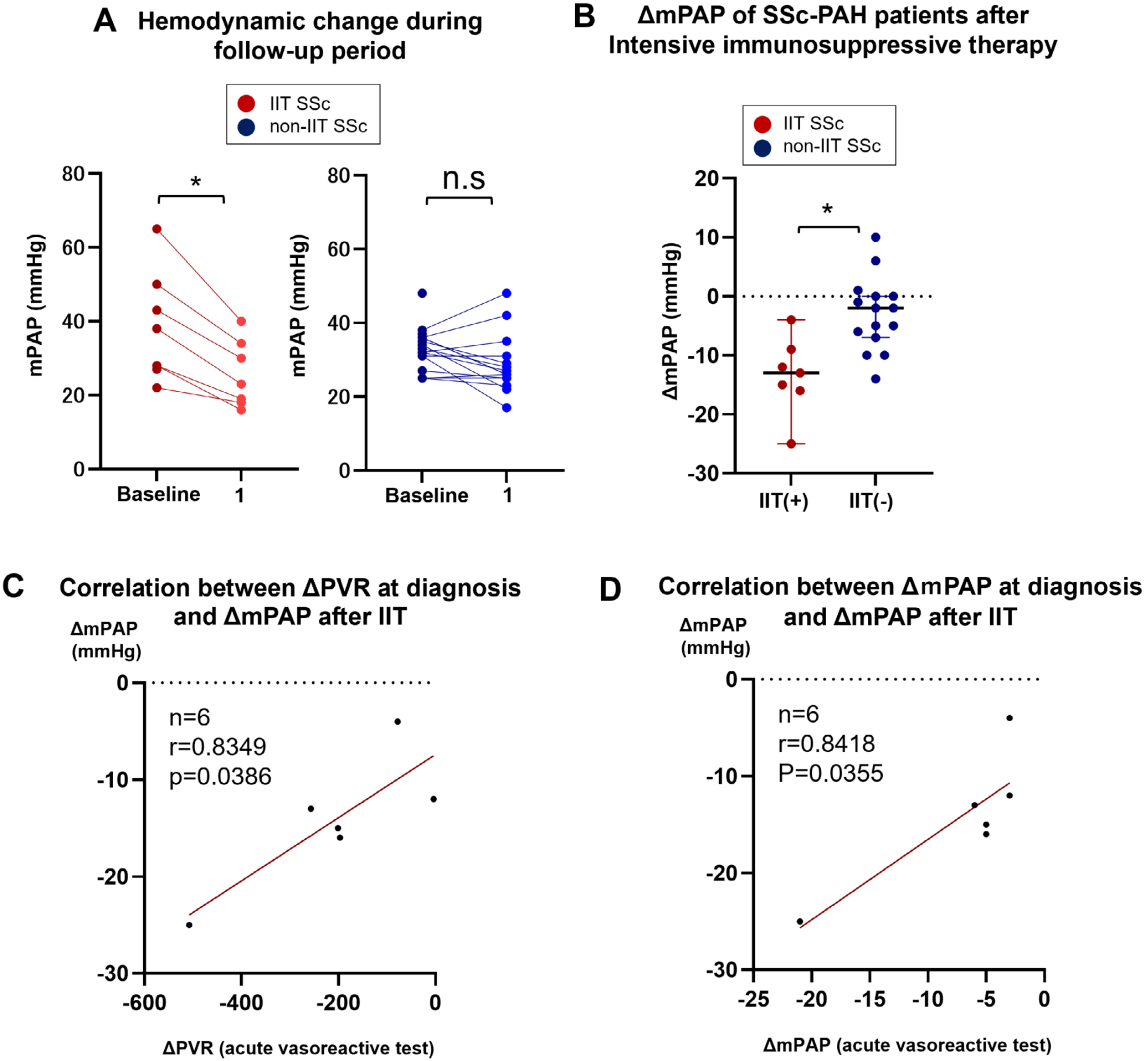
Effects of IIT on pulmonary hemodynamics in patients with systemic sclerosis. (A) Effect of intensive immunosuppressive therapy (IIT) on pulmonary hemodynamics in patients with pulmonary arterial hypertension associated with systemic sclerosis. The red bars represent the IIT group, while the blue bars represent the non‐IIT group. (B) Change in the mean pulmonary artery pressure (ΔmPAP) in patients with systemic sclerosis 1 year after intensive immunosuppressive therapy (IIT group) and no IIT (non‐IIT group). (C) Correlation between the decrease in pulmonary vascular resistance (PVR) during the acute vasoreactive test at diagnosis and the improvement in the mPAP after IIT in the IIT‐SSc‐PAH group. (D) Correlation between the decrease in the mPAP during the acute vasoreactive test at diagnosis and the improvement in the mPAP after IIT in the IIT‐SSc‐PAH group. The results are presented as the mean ± SEM. Statistical comparisons were performed using paired *t*‐tests, two‐tailed Student's *t*‐tests, or Welch's *t*‐tests as appropriate. The significance levels are indicated as follows: *p < 0.05, **p < 0.01.

While previous studies have reported that IIT is ineffective in patients with SSc‐PAH [4, 9], they were marred by the small sample size. This study included 27 patients with SSc‐PAH, of whom 9 received IIT and 18 did not receive IIT. The baseline characteristics of these patients are shown in Table 2, which showed blood collection data, interstitial lung disease comorbidity, pulmonary hemodynamics, disease duration, and disease subtype were comparable between the IIT‐SSc‐PAH and non‐IIT‐SSc‐PAH groups. The proportion of patients with other CTDs was significantly higher in the IIT‐SSc‐PAH group than that in the non‐IIT group. Additionally, although no SSc‐PAH patients met the positive criteria for the acute vasoreactivity test, the IIT‐SSc‐PAH group showed a significantly greater reduction in mPAP and PVR during the acute pulmonary vasoreactivity test (Table 2). Notably, the improvement in mPAP was significantly more pronounced in the IIT‐SSc‐PAH group (Figure 4A,B). Furthermore, changes in the PVR and mPAP during the vasoreactive test at diagnosis were strongly correlated with mPAP improvement following IIT in the IIT‐SSc‐PAH group (PVR: *r* = 0.835, *p* = 0.0386; mPAP: r = 0.842, *p* = 0.0355) (Figure 4C,D), but no such improvement was apparent in the non‐IIT group (Figure S1A,B).

**TABLE 2:** Baseline characteristics of patients with pulmonary arterial hypertension associated with systemic sclerosis treated with or without intensive immunosuppressive therapy.

	IIT (*n* = 9)	Non‐IIT (*n* = 18)	*p*
Age (years)	59.7 ± 8.8	64.2 ± 10.7	0.172
Female (%)	8 (88.9)	13 (72.2)	0.326
Follow‐up period (month)	36.0 (15.5–70.0)	51.0 (30.0–74.0)	0.395
Diffuse type of SSc	2 (22.2)	5 (27.8)	0.756
Limited type of SSc	7 (77.8)	13 (72.2)	0.756
Anti‐centromere	4 (44.4)	6 (33.3)	0.573
Anti‐Scl70	1 (11.1)	1 (5.6)	0.603
Time from diagnosis of CTD to diagnosis of PAH	24.0 (0.0–132.0)	90.0 (0.0–80.5)	0.526
Disease duration at end of follow‐up	136.0 (81.0–300)	120.0 (75.5–204.0)	0.554
Simultaneous diagnosis of PAH and CTD	3 (33.3)	6 (35.3)	0.920
ILD	3 (33.3)	10 (55.6)	0.276
Underlying CTDs overlapped with SSc (%)			
Any CTDs overlapped with SSc	4 (44.4)	2 (11.1)	0.050
SLE	1 (11.1)	0 (0.0)	0.149
SjS	3 (33.3)	2 (11.1)	0.161
Medications after 1 year (*n*, %)			
PDE5i or sGC stimulator	4 (44.4)	16 (88.9)	0.013
ERA	3 (33.3)	10 (55.6)	0.276
Oral PGI_2_ analogs	4 (44.4)	5 (27.8)	0.387
Treprostinil or epoprostenol	0 (0.0)	1 (5.6)	0.471
Mono therapy	3 (33.3)	7 (38.9)	0.778
Dual therapy	4 (44.4)	8 (44.4)	1.000
Triple therapy	0 (0.0)	3 (16.7)	0.050
Baseline measurement			
Hb (g/dL)	13.2 ± 1.4	12.4 ± 2.1	0.358
Creatinine (mg/dL)	0.6 ± 0.1	0.8 ± 0.4	0.298
CRP (mg/dL)	0.0 (0.0–0.1)	0.0 (0.0–0.1)	0.967
BNP (pg/mL)	127.3 (7.3–441.6)	75.0 (26.3–454.5)	0.602
NYHA			
I	1 (11.1)	2 (11.1)	1.000
II	4 (44.4)	9 (50.0)	0.785
III	4 (44.4)	6 (33.3)	0.573
IV	0 (0.0)	2 (11.1)	0.298
Pulmonary hemodynamic			
mPAP (mmHg)	35.9 ± 14.5	33.8 ± 7.2	0.898
PVR (dyne s/cm^2^)	689.5 ± 480.5	480.7 ± 246.1	0.520
CI (L/min/m^2^)	2.3 ± 0.4	2.4 ± 0.3	0.571
PAWP (mmHg)	7.5 ± 2.9	8.8 ± 8.0	0.965
RAP (mmHg)	4.7 ± 2.2	4.9 ± 4.3	0.875
SvO_2_ (%)	68.8 ± 6.7	65.7 ± 8.0	0.365
Hemodynamic change after the acute vasoreactive test			
ΔmPAP (mmHg)	−6.9 ± 6.3	−3.0 ± 3.8	0.029
ΔPVR (dyne s/cm^2^)	−189.1 ± 166.0	−50.3 ± 86.4	0.034
ΔCI (L/min/m^2^)	−0.06 ± 0.39	−0.08 ± 0.38	1.000

*Note:* Continuous variables are expressed as the mean ± SD, except for the follow‐up period. The eGFR and BNP levels are expressed as medians with interquartile ranges. The parameters were compared using the Mann–Whitney *U* test.

Abbreviations: BNP, brain natriuretic peptide; CI, cardiac index; CTD, connective tissue disease; ERA, endothelin receptor antagonist; ILD, interstitial lung disease; MCTD, mixed connective tissue disease; PAH, pulmonary arterial hypertension; PAP, pulmonary artery pressure; PAWP, pulmonary arterial wedge pressure; PDE5i, phosphodiesterase inhibitor; PGI2, prostacyclin; PVR, pulmonary vascular resistance; RAP, right arterial pressure; sGC, soluble guanylate cyclase; SjS, Sjögren syndrome; SLE, systemic lupus erythematosus; SSc, systemic sclerosis; SvO_2_, mixed venous oxygen saturation.

## 4 Discussion

The salient findings of this study were as follows: (1) the favorable effect of IIT on both prognosis and hemodynamics in CTD‐PAH was sustained over prolonged periods without the need for additional IIT due to worsening of PH, and (2) IIT could be potentially effective in patients with SSc‐PAH. These results indicate that IIT can be effective for patients with CTD‐PAH in the long term.

### 4.1 Long‐Term Benefits of IIT: CTD‐PAH

The first key finding of this study is that IIT not only improved pulmonary hemodynamics, but also sustained these improvements over an extended follow‐up and yielded a positive effect on prognosis. While earlier studies have confirmed that IIT benefits patients with CTD‐PAH, they primarily focused on short‐term outcomes (improvement in hemodynamics or WHO functional class after 3 months to 1 year) [7, 8, 18, 19], without examining the persistence of pulmonary hemodynamic improvements. This fostered uncertainty regarding the durability of the effects of IIT. In this study, sustained improvements in both prognosis and pulmonary hemodynamics were confirmed over a prolonged median follow‐up of 5.3 years. Importantly, no patient required repeat IIT owing to worsening of PH, indicating that once pulmonary hemodynamics improved, the effects were sustained. Previous studies have reported an infection incidence of approximately 10%–15% with IIT [20], which is consistent with our findings. This study further revealed that adverse events and adverse event‐related mortality did not significantly increase during long‐term follow‐up after IIT. These findings suggest that IIT is both effective and safe over the long term for patients with CTD‐PAH. However, in this study, the treatment of patients with CTD‐PAH was individualized rather than randomized. Further randomized studies are needed to confirm these findings while minimizing individual bias.

### 4.2 Effectiveness of IIT: SSc‐Associated PAH

The second key finding was that IIT was effective in improving pulmonary hemodynamics in some patients with SSc‐PAH. Previous reports have suggested that IIT was ineffective for SSc‐PAH, leading to its exclusion from the ESC/ERS guidelines [4, 9]. However, these studies incorporated a limited sample size, and recent case reports indicate that IIT can improve pulmonary hemodynamics in patients with SSc‐PAH [21, 22]. Additionally, a larger study showed that immunosuppressive therapy with rituximab, which targets B cells, enhances exercise capacity in these patients [23].

In this study, IIT improved pulmonary hemodynamics in patients with SSc‐PAH more effectively than previously reported [9]. Two primary factors may explain this discrepancy. First, our cohort included a higher proportion of patients with overlapping CTDs other than SSc. Previous studies have shown that SSc‐PAH patients with lupus tend to respond better to IIT [22, 24]. Second, the IIT‐SSc‐PAH group exhibited significantly greater reductions in mPAP and PVR during the acute pulmonary vasoreactivity test compared to the non‐IIT‐SSc‐PAH group. Previous studies have reported a lower positive response rate in SSc‐PAH than in idiopathic PAH, with decreased vasoreactivity being correlated with disease progression [25, 26, 27, 28]. Importantly, the analysis demonstrated a positive correlation between reductions in PVR and mPAP during the acute vasoreactivity test, and subsequent improvements in mPAP following IIT. This suggests that patients with SSc‐PAH with better hemodynamic responses may exhibit less severe vascular remodeling, making them more likely to benefit from IIT. Although previous studies did not report data on overlap with other CTDs or the results of the acute vasoreactivity test [9]. Our findings suggest that some SSc‐PAH patients may retain pulmonary vasoreactivity. This potential selection bias might have contributed to the favorable outcomes observed in our study. These findings indicate that a subset of patients with SSc‐PAH may respond favorably to IIT, although further research with larger cohorts is needed to confirm these outcomes owing to the small sample size.

### 4.3 Limitations

The present study has several limitations. First, this was a retrospective cohort study conducted at a single center. Owing to these characteristics, the differences in the patient characteristics between the groups and the presence of multiple confounding factors might have influenced the results. Therefore, the findings regarding the effectiveness of IIT need to be validated in a larger, multicenter study. Second, the treatment of patients with CTD‐PAH was individualized rather than randomized, based on pulmonary hemodynamics and comorbidities. Third, the use of IIT was determined at the physicians' discretion, and the baseline characteristics of the two groups differed considerably. These differences may have introduced residual confounding, and thus the possibility of selection bias influencing the results cannot be excluded. Fourth, isolating the effect of IIT was challenging, as patients with CTD‐PAH received pulmonary vasodilators in conjunction with IIT. Finally, although IIT appeared effective in reducing PH‐related death, the small number of events may have resulted in an overestimation of the effect. Therefore, further studies with larger sample sizes are warranted to confirm these findings.

## 5 Conclusions

These findings suggest that IIT may lead to sustained improvements in pulmonary hemodynamics and better long‐term outcomes in patients with CTD‐PAH, including potential benefits in patients with SSc‐PAH.

## Author Contributions

K.Y., N.Y., and S.Y. contributed to the conceptualization of the study. T.S., S.Y., Y.Y., N.C., K.K., H.S., N.K., K.N., S.T., and T.I. supervised the study and performed data acquisition. S.M. performed statistical analysis. All authors read and approved the final manuscript. K.Y. takes responsibility for the content of the manuscript, including the data and analyses.

## Conflicts of Interest

The authors declare no conflicts of interest.

## Supporting information


**Appendix S1:** apl70431‐sup‐0001‐AppendixS1.docx.

## Data Availability

The data that support the findings of this study are available on request from the corresponding author. The data are not publicly available due to privacy or ethical restrictions.
