# Supplementary material for: Long‐Term Prognostic and Hemodynamic Outcomes of Intensive Immunosuppressive Therapy in Patients With Pulmonary Arterial Hypertension Associated With Connective Tissue Disease
Source: Int J Rheum Dis. 2025 Oct 9;28(10):e70431. doi: 10.1111/1756-185X.70431 (PMC12509181; doi:10.1111/1756-185X.70431)

**Supplemental File**

**Long-Term Prognostic and Hemodynamic Outcomes of Intensive Immunosuppressive Therapy in Patients with Pulmonary Arterial Hypertension Associated with Connective Tissue Disease**

Kaito Yamada, Nobuhiro Yaoita, Taijyu Satoh, Saori Yamamoto, Yusuke Yamada, Naoki Chiba, Kohei Komaru, Haruka Sato, Nobuhiro Kikuchi, Hideaki Suzuki, Kotaro Nochioka, Shunsuke Tatebe, Satoshi Miyata, Tomonori Ishii, Satoshi Yasuda

| **Table S1. Study Enrollment Period and Follow-up in Patients with Pulmonary Arterial Hypertension Associated with Connective Tissue Disease** | | | | | | |
| --- | --- | --- | --- | --- | --- | --- |
|  |  |  |  |  |  |  |
|  |  | IIT (n=41) |  | Non-IIT (n=28) |  | p value |
| Study enrollment period (%) |  | 59.7 ±8.8 |  | 0 (0.0) |  | 0.143 |
| 2001-2005 |  | 3 (7.3) |  | 3 (10.7) |  | 0.461 |
| 2006-2010 |  | 7 (17.1) |  | 8 (28.6) |  | 0.698 |
| 2011-2015 |  | 10 (24.4) |  | 17 (60.7) |  | 0.436 |
| 2016- |  | 21 (51.2) |  | 4 (14.3) |  | 0.564 |
| Lost to follow up |  | 4 (9.8) |  | 0 (0.0) |  | 0.143 |

.

The parameters were compared using the Pearson's chi-square test test.

**Figure S1. Correlation between the decrease in the PVR (A) and mPAP (B) in the acute vasoreactive test at diagnosis and the improvement in the mPAP after 1 year in the the non-IIT-SSc-PAH group**


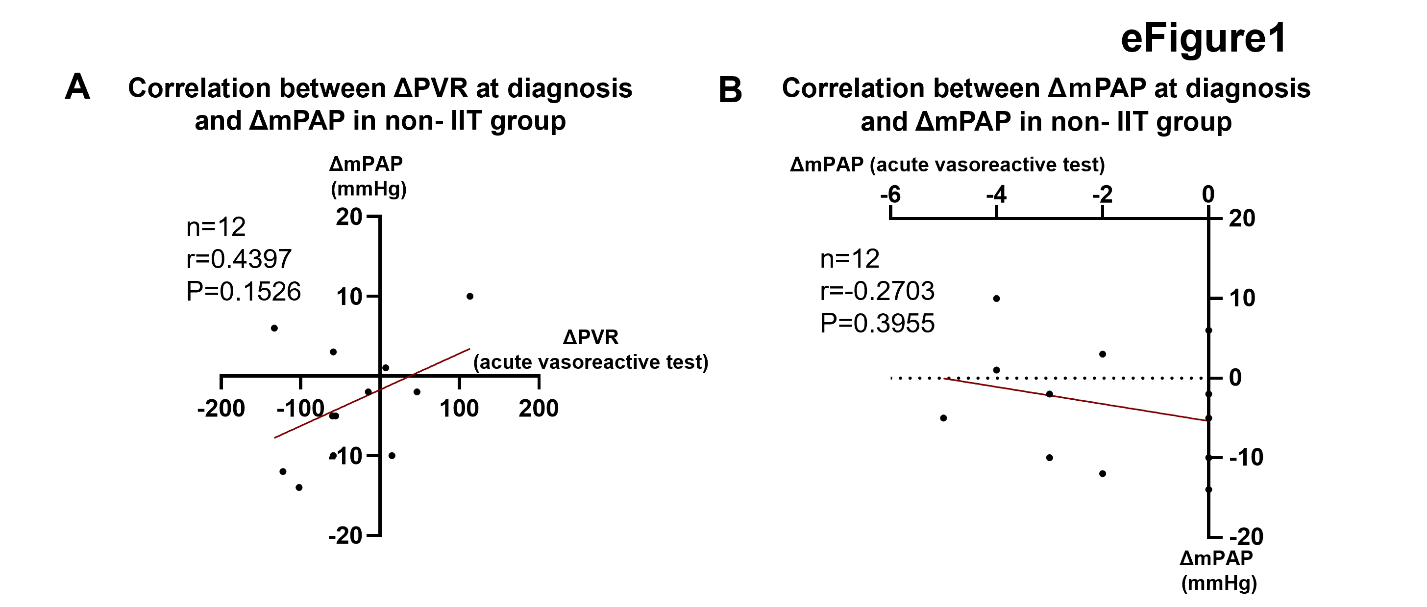

Supplement: Supplementary file 1 — Appendix S1: apl70431‐sup‐0001‐AppendixS1.docx. [file APL-28-e70431-s001.docx]
